# Development and validation of a nursing practice scale for supporting fertility preservation decision-making in women with cancer

**DOI:** 10.1016/j.apjon.2025.100808

**Published:** 2025-10-24

**Authors:** Mikako Yoshihara, Kazuaki Tanabe, Chie Teramoto, Hiroyuki Sawatari, Ruxin Lei, Hisae Nakatani

**Affiliations:** Department of Perioperative and Critical Care Management, Graduate School of Biomedical and Health Sciences, Hiroshima University, Hiroshima, Japan

**Keywords:** Decision-making, Nursing care, Psychometrics, Oncology nursing, Patient-centered care, Validation study

## Abstract

**Objective:**

With increasing attention to fertility preservation (FP) among women with cancer, nursing support for FP decision-making remains insufficient and lacks standardization. Nurses often face challenges such as limited knowledge, inconsistent practices, and the absence of structured decision-support frameworks. This study aimed to develop and psychometrically validate a scale to assess nursing practices that support FP decision-making in women with cancer.

**Methods:**

The study comprised two phases: scale development and psychometric evaluation. In Phase 1, an initial item pool was generated through a literature review, expert consultation, and content validity assessment. In Phase 2, a cross-sectional survey was conducted among 600 nurses, who were divided into exploratory factor analysis (EFA; *n* ​= ​282) and confirmatory factor analysis (CFA; *n* ​= ​318) groups. Construct validity was examined using EFA and CFA, and criterion-related, convergent, and discriminant validity were evaluated. Reliability was tested using Cronbach's α.

**Results:**

The preliminary 22-item, four-factor scale demonstrated strong content validity. Subsequent analyses produced a refined 12-item scale comprising three factors: (1) providing professional support for patients’FP decision-making, (2) facilitating communication between patients and families, and (3) promoting system-based and team-oriented FP support. The scale demonstrated excellent internal consistency (Cronbach's α ​= ​0.921). CFA indicated good model fit (Comparative Fit Index ​= ​0.969, Tucker–Lewis Index ​= ​0.960, Root Mean Square Error of Approximation ​= ​0.077; *P* ​< ​0.001).

**Conclusions:**

This study developed a reliable and valid 12-item, three-factor scale to assess nursing practices supporting FP decision-making in women with cancer. The scale may serve as a useful tool for evaluating and enhancing patient-centered FP care in oncology nursing practice.

## Introduction

The International Agency for Research on Cancer estimated that in 2022, 20 million people worldwide were newly diagnosed with cancer and 9.7 million died of cancer-related events.^1^ Recent advances in diagnostics and multimodal treatments have improved the prognosis of patients with cancer.^2^ Oncofertility, a multidisciplinary field that bridges oncology and reproductive medicine and addresses fertility-related issues in patients undergoing cancer treatment, has also been developed alongside the multimodal treatment approaches.^3^ However, cancer treatment can impair patients’ reproductive ability. For example, chemotherapy and radiation treatments have been shown to impair ovarian function, leading to fertility loss in women.^4^^,^^5^ Previous studies have reported that appropriate interventions before and after cancer treatment, such as reproductive technologies, could preserve fertility in women with cancer.^6^^,^^7^

A prior study reported that many patients with cancer experienced psychological distress and/or confusion when making decisions about fertility preservation (FP).^8^ Since inadequate or delayed information provision can lead to missed opportunities for FP,^9^ healthcare professionals—particularly nurses, who are usually the first to interact with patients—should provide timely decision-making support. However, critical gaps exist regarding support for FP in nursing practice.^10^ Nurses face challenges such as limited education, lack of time, poor interprofessional collaboration, and insufficient knowledge about FP.^11^ According to previous research, nurse-led decision support for FP has been reported; however, concerns have been raised regarding its feasibility due to variability in nurses' competencies, role ambiguity, and potential overlaps with physicians’ responsibilities.^12^ These conflicting findings underscore the importance of defining nursing roles and enhancing interprofessional collaboration regarding FP.^13^

The Ministry of Health, Labour and Welfare in Japan launched national research programs to promote FP therapy in adolescents and young adults with cancer. This underscores the importance of multidisciplinary collaboration among healthcare professionals, and of establishing a comprehensive support system for patient consultation.^14^ Several studies have examined the needs and experiences of women with cancer regarding support for decision-making on FP.^15^ Women with cancer frequently experience psychological distress when making decisions about FP during treatments.^16^^,^^17^ Consequently, when nurses do not provide appropriate decision-making support, some of them decline or lose the opportunity for FP.^18^ While many studies have reported on the needs and experiences of women with cancer regarding FP decisions,^19^^,^^20^ standardized assessment tools for evaluating nursing support for FP are unavailable. Existing scales focus on knowledge or attitudes toward FP among nurses, overlooking aspects of support for decision-making.^16^ Therefore, a theoretical framework is necessary to ensure consistent, evidence-based, and person-centered support. Accordingly, the present study aimed to develop a scale to measure nursing practices that support decision-making for FP in women with cancer, and examine its reliability and validity. The present study used the Shared Decision-Making (SDM) model, which promotes collaborative decision-making between patients and healthcare providers and is suitable for preference-sensitive care such as FP.^21^^,^^22^

## Methods

This study consisted of the following two steps: developing the initial scale, “10.13039/100028824Nursing Practices to Support Decision-Making for FP in Women with Cancer,” and evaluating its reliability and validity.

### Step 1: developing the initial scale

#### Literature review and thematic coding

As a first step in developing the initial version of the scale, a literature review was conducted to clarify nursing practices that support FP decision-making in women with cancer. In February 2022, Japanese literature was reviewed through the “ICHUSHI web version” and “CiNii Articles” database; English literature was searched using “PubMed” and “Web of Science.” The literature review was limited to publications in both English and Japanese, enabling the inclusion of international perspectives and best practices, as well as consideration of cultural and healthcare system differences across countries. The keywords used for the review were “patient with cancer,” “fertility preservation,” and “nursing.” As a second step, researchers gathered expert feedback on the initial items from nurses specializing in the care of women with cancer, university faculty members, and graduate nursing students.

Selection criteria for the experts included having > 5 years of clinical or educational experience caring for women with cancer, and consenting to participate in this study. The criterion of ≥ 5 years of clinical and/or educational experience for expert panel members was based on theoretical and empirical grounds. First, Benner's theory of nursing expertise suggested that approximately 5 years of clinical experience are needed for nurses to be considered “proficient” or “expert,” which is essential for acquiring intuitive clinical judgment and advanced practice skills regarding specialized nursing fields.^23^ Second, a Delphi study showed core competencies for advanced oncology nursing education and required all expert panel members to have at least 5 years of professional experience to ensure the credibility of responses.^24^^,^^25^ Based on their feedback, the initial version of the scale was revised to confirm its relevance in assessing support for decision-making regarding FP in women with cancer.

Accordingly, several items were revised to improve clarity and ease of response. For example, the original item “I consider the patient's feelings of uncertainty” was modified to “I support the patient's emotional state, considering that the patient may be unstable and anxious.” Such revisions enhanced the clarity of the items throughout the scale, ensuring that the revised wording better reflected nursing practices and was easier for respondents to understand.

#### Creation of the draft version of the scale and content validity

Content validity was assessed using item-level content validity index (I-CVI) and scale-level content validity index (S-CVI). Six experts evaluated the content and face validities of the scale during a pilot test. The criterion of ≥ 5 years of clinical and/or educational experience for expert panel members was based on both theoretical and empirical grounds. Two of the six experts were the same for the literature review and thematic coding process. The experts were required to meet at least one of the following criteria: having a master's degree or higher, being a nurse or midwife with > 5 years of experience, or having experience in scale development and validity verification. On the scale, the evaluated points were as follows: appropriately worded, easy to understand and answer, and free from content deviation. The items were revised through repeated discussions based on the expert opinions. In this study, the I-CVI and S-CVI/Universal Agreement (S-CVI/UA) of ≥ 0.90 and ​≥ ​0.80, respectively, were considered as having high content validity.^26^ Six experts evaluated each item on the scale using a four-point Likert scale ranging from “appropriate” to “inappropriate.” Certified oncology nurse navigators with experience in patient consultations at cancer-designated hospitals and academic experts in nursing science provided supervision to enhance the validity of the qualitative analysis of the initial items.

#### Examination of the content validity of the draft scale

A pilot test was used to evaluate the clarity and understandability of each item. Four participants were included in the examination of the content validity of the draft scale. Based on previous research, this number was deemed sufficient to identify issues with phrasing and comprehension.^27^ One nurse who provided care to women with cancer and three midwives were asked to complete a questionnaire to evaluate the clarity and understandability of each item. They were also asked about any ambiguities or difficulties in responding to the questions. Patients were not consulted prior to the scale development.

### Step 2: evaluation of the reliability and validity of the scale

#### Study design

The second step involved a cross-sectional questionnaire-based study targeting 450 institutions designated as part of Japan's national cancer control framework, in accordance with the Headquarters of Cancer Control of the Ministry of Health, Labour, and Welfare. In particular, the study periods for exploratory factor analysis (EFA) and confirmatory factor analysis (CFA) were from February to April 2023 and January to April 2024, respectively. Data were obtained using a self-administered questionnaire without identifiable information.

#### Participants

This study included nurses who had experience in providing consultation or nursing care for women with cancer at cancer consultation support centers, inpatient wards, or hospital outpatient institutions, and those who were employed at institutions designated as part of Japan's national cancer control framework, in accordance with the Headquarters of Cancer Control of the 10.13039/100009647Ministry of Health, Labour, and Welfare. Nurses without experience in caring for women with cancer, as well as those on maternity leave, sick leave, or absent from clinical practice for other reasons during the survey period, were excluded. In addition to the inclusion and exclusion criteria, questionnaires with any missing responses on the scale items were excluded from the analysis.

Participants were recruited using a convenience sampling method through nursing department representatives (e.g., chief nurses or nurse managers) at institutions designated as part of Japan's national cancer control framework in accordance with the Headquarters of Cancer Control of the Ministry of Health, Labour and Welfare, Japan The representatives distributed questionnaires to eligible nurses who were working in oncology wards or outpatient units, and those with experience caring for women with cancer. Participation was voluntary and anonymous, and responses were collected either by mail, using sealed envelopes, or a secure web-based survey system to ensure participant confidentiality. A reminder was sent by postal mail to increase the response rate and minimize bias associated with non-response.

#### Measures

This study's questionnaire included basic characteristics, a draft version of the scale for assessing nursing practice that supports FP decision-making in women with cancer, and a scale for evaluating criterion-related validity. Data on participants' basic characteristics included age, years of nursing experience, years of experience in the current workplace, job title, professional qualifications, presence of a reproductive medicine department to provide FP treatments, experiences in providing consultation to women with cancer within the past 2 years, and training experience on FP. A five-point Likert scale (“5 ​= ​mostly,” “4 ​= ​occasionally,” “3 ​= ​neither,” “2 ​= ​rarely,” and “1 ​= ​never”) was used for the scale assessing nursing practice that supports FP decision-making in women with cancer. The scale consists of 22 items, with total scores calculated by summing the scores of all items. Specifically, the total scores range from 22 to 110 points, with higher scores indicating higher levels of nursing practice.

#### Statistical analysis

Data are presented as means ​± ​standard deviation (SD) and frequencies (%). Normality of continuous variables was assessed using the Shapiro–Wilk test. Group comparisons were conducted using the independent samples *t* test for variables that met the assumption of normality, and the non-parametric Mann–Whitney *U* test for those that did not. This approach ensured the use of appropriate statistical methods based on data distribution. The χ^2^ test was used to compare categorical variables between the two groups.

Based on prior studies, the required sample sizes for EFA and CFA were estimated to be at least three times the number of scale items and a minimum of 100 and 200 participants, respectively.^28^, ^29^, ^30^ The sample size aligns with recent trends in factor analysis research.^31^ Considering the expected response rate and potential missing data, the effective response rate was estimated to be approximately 30% to 40%.^32^^,^^33^ The required number of survey distributions in the EFA and CFA groups was more than 350 and 670, respectively.

The scale's reliability was assessed for item analysis by calculating ceiling and floor effects, inter-item correlations, and item-total (I-T) correlations. Ceiling and floor effects were defined as the mean plus one SD and the mean minus one SD, respectively. Inter-item correlations exceeding *r* ​> ​0.75 were considered strongly correlated and potentially influential on the results.^34^ An exclusion criterion of *r* ​< ​0.30 was adopted for I-T correlations, reflecting insufficient internal consistency between the item and total scale scores.^35^ Good–poor (GP) analysis was performed using the top and bottom 25% of respondents as cut-off points. The scores for each developed item were compared between the high-scoring (*n* ​= ​70) and low-scoring (*n* ​= ​70) groups. Cronbach's α coefficients were calculated for the entire scale and its individual factors to assess reliability, with a threshold of > 0.70 considered acceptable.^36^

For the EFA, the Kaiser–Meyer–Olkin (KMO) measure of sampling adequacy and Bartlett's test of sphericity were used to assess the adequacy and appropriateness of the sample for factor analysis. Bartlett's test was significant when the KMO value exceeded 0.5 (*P* ​< ​0.001), indicating sufficient sampling adequacy and suitability for factor analysis.^37^ The maximum likelihood method with promax rotation was used for EFA. Both scree plot inspection and parallel analysis were conducted to determine the appropriate number of factors in the EFA. Promax (oblique) rotation was employed in the present study since the theoretical constructs underlying the scale (i.e., provision of information, emotional support, and communication regarding FP decision-making) were conceptually expected to be correlated rather than independent.

This approach allows for the estimation of inter-factor correlations, which is appropriate given the multidimensional and overlapping nature of nursing practices in this context. Items with factor loadings ≤ 0.4 were deleted from the model.^38^ After identifying the factor structure, each factor was named as logically and conceptually appropriate.

CFA was used to confirm the scale's factor structure and validity. Model fit was evaluated using the Goodness of Fit Index (GFI), Adjusted Goodness of Fit Index (AGFI), Tucker–Lewis Index (TLI), Comparative Fit Index (CFI), and Root Mean Square Error of Approximation (RMSEA). Acceptable model fit was defined as GFI > 0.90, TLI > 0.90, CFI > 0.90, and RMSEA < 0.08.^37^ Criterion-related validity of the developed scale was also examined.

The association between the newly developed scale and the “10.13039/100028824Nursing Support for Patient Treatment Decision-Making Scale,” which has been previously validated and demonstrated reliability and validity,^39^ was assessed to evaluate the criterion-related validity of the developed scale using Spearman's rank correlation coefficient since the variables involved non-10.13039/100014230Gaussian distribution. Convergent validity was assessed using Composite Reliability (CR) and Average Variance Extracted (AVE). The criteria for convergent validity were CR ​> ​0.70, AVE > 0.50, and CR ​> ​AVE.^36^ We applied the criterion proposed by Fornell and Larcker to assess discriminant validity,^40^ verifying that the square root of the AVE (√AVE) for each construct exceeded the correlations with other constructs. Additionally, discriminant validity was assessed using the Heterotrait-Monotrait (HTMT) ratio.

All data were analyzed using IBM SPSS Statistics for Windows, version 29 (IBM Corp., Armonk, NY, USA) or IBM SPSS AMOS (IBM Corp., Armonk, NY, USA) version 29.

## Results

### Step 1: determining the initial scale

Regarding content validity, the I-CVI and S-CVI/UA exceeded 0.98 and 0.91, respectively. Four items were revised based on expert feedback. After the revisions, all items achieved an I-CVI and S-CVI/UA of 1.00.

10.13039/100028824Nursing practices supporting decision-making in women with cancer were categorized into the following four domains for the qualitative analysis of the initial items: appropriate support for patients regarding FP, support based on a coordinated healthcare system, support provided by professionals with specialized knowledge of oncofertility, and appropriate support for families and partners concerning FP. In addition to these domains, 22 items served as the foundation for developing the initial draft of a scale assessing nursing practice that supports FP decision-making in women with cancer. The pilot test showed that the 22 items were comprehensible, without any issues regarding respondent burden.

### Step 2: evaluation of the reliability and validity of the scale

**Fig. 1:**
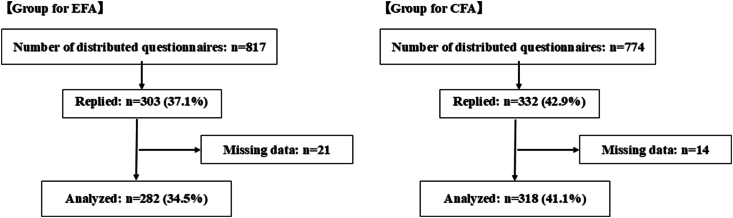
**Participant recruitment and selection flowchart**. EFA, exploratory factor analysis; CFA, comparative factor analysis.

Questionnaires were distributed to 817 nurses in the EFA group, and 282 valid responses from participants without missing data on the nursing practice scale items were analyzed (valid response rate: 34.5%). In the CFA group, questionnaires were distributed to 774 nurses and 318 valid responses were analyzed (valid response rate: 41.1%) (Fig. 1). In addition to the inclusion and exclusion criteria, questionnaires with any missing responses on the nursing practice scale items were excluded from the analysis.

As shown in Table 1, nurses in the EFA group were significantly older than those in the CFA group (43.0 ​± ​9.1 vs. 41.3 ​± ​10.2 years, *P* ​= ​0.012). The Shapiro–Wilk test indicated that age was normally distributed in the EFA group (W ​= ​0.991, *P* ​= ​0.094) but not in the CFA group (W ​= ​0.978, *P* ​< ​0.001). Meanwhile, no significant differences were observed in the job title, period of working as a nurse, or the period at the current department. Regarding years of nursing experience, normality was supported in the EFA group (W ​= ​0.991, *P* ​= ​0.098) but not in the CFA group (W ​= ​0.982, *P* ​< ​0.001). The experience in providing counseling for women with cancer in the past 2 years was 36.5% and 24.1% in the EFA and CFA groups, respectively (*P* ​= ​0.002). For nurses with other professional qualifications, the EFA group had more qualifications than the CFA group (48.2 vs. 37.1%; *P* ​= ​0.006).

**Table 1:** Participants' and hospitals’ characteristics.

Characteristics	EFA (*n* ​= ​282)	CFA (*n* ​= ​318)	*P*-value
**Age, years**	43.0 ​± ​9.1	41.3 ​± ​10.2	0.012
**Working periods as a nurse, years**	20.3 ​± ​9.2	17.9 ​± ​9.3	0.054
**Working periods in the current department, years**	5.6 ​± ​4.6	5.4 ​± ​4.9	0.151
**Presence of fertility department, *n* (%)**			
Exist	69 (25.0)	118 (38.4)	< 0.001
Absent	207 (75.0)	189 (61.6)	
**Job title, *n* (%)**			
Chief nursing officer	24 (8.5)	22 (6.9)	0.243
Assistant chief nursing officer	65 (23.1)	61 (19.2)	
Stuff	190 (67.6)	228 (71.7)	
Other	2 (0.7)	7 (2.2)	
**Belonging department, *n* (%)**			
Ward	120 (42.7)	148 (46.7)	< 0.001
Outpatient department	84 (29.9)	125 (39.4)	
Cancer consultation and support center	45 (16.0)	27 (8.5)	
Other	32 (11.4)	17 (5.4)	
**Other types of professional qualifications, *n* (%)**			
Exist	136 (48.2)	118 (37.1)	0.006
Absent	146 (51.8)	200 (62.9)	
**Experience of consultants about fertility preservation, *n* (%)**			
Experienced	103 (36.5)	76 (24.1)	0.002
Never	179 (63.5)	240 (75.9)	
**Experience of training about fertility preservation, *n* (%)**			
Experienced	123 (43.6)	111 (35.0)	0.031
Never	159 (56.4)	206 (65.0)	

EFA, exploratory factor analysis; CFA, comparative factor analysis.

Missing data: Presence of fertility department: *n* ​= ​17, Job title: *n* ​= ​1, Belonging department: *n* ​= ​2, Experience of consultants about fertility preservation: *n* ​= ​2, and Experience of training about fertility preservation: *n* ​= ​1.

Regarding item analysis, the I-T correlation coefficients ranged from 0.419 to 0.872. No items had internal consistency in the scale, and no ceiling effects were observed. Two items (8 and 10) exhibiting floor effects were removed (Table 2). For item-to-item correlations, six items were removed due to high correlation values (#1: *r* ​= ​0.834, #3: *r* ​= ​0.762, #5: *r* ​= ​0.806, #13: *r* ​= ​0.876, #20: *r* ​= ​0.766, and #21: *r* ​= ​0.814). Fourteen items were included in the model. The GP analysis showed that the higher-scoring group had significantly greater scores for each item than the lower-scoring group (*P* ​< ​0.001 for all items) (Table 2).

**Table 2:** Initial item analysis of the scale for assessing nursing practices supporting fertility preservation decision-making in women with cancer.

Item		Mean	SD	Ceiling effect	Floor effect	I-T correlation	*P*-value (GP analysis)
1	I ensure that patients can easily ask nurses questions.	3.82	0.975	4.795	2.845	0.451^a^	< 0.001^a^
2	I support the patient's emotional state, considering that the patient may be unstable and anxious.	3.87	0.941	4.811	2.929	0.469^a^	< 0.001^a^
3	I confirm the patients' wishes regarding fertility preservation when I collect their information.	3.00	1.299	4.299	1.701	0.823^a^	< 0.001^a^
4	I gather information about the presence of the patient's partner and their relationship early after the cancer diagnosis.	3.38	1.176	4.556	2.204	0.647^a^	< 0.001^a^
5	I confirm the patients' understanding of fertility preservation.	2.64	1.281	3.921	1.359	0.864^a^	< 0.001^a^
6	I confirm whether a difference exists between actual fertility preservation and the fertility preservation recognized by the patient.	2.59	1.339	3.929	1.251	0.872^a^	< 0.001^a^
7	I appropriately share patient information with the cancer specialist who is treating them.	3.43	1.186	4.616	2.244	0.596^a^	< 0.001^a^
8	I share patient information with the reproductive medicine specialist as needed.	1.92	1.143	3.063	0.777	0.573^a^	< 0.001^a^
9	I appropriately share patient information with the nurses who belong to the same department.	3.48	1.157	4.637	2.323	0.419^a^	< 0.001^a^
10	I share patient information with the reproductive medicine nurse as needed.	1.86	1.173	3.033	0.687	0.545^a^	< 0.001^a^
11	I share patient information regarding fertility preservation with the medical team involved in fertility preservation.	2.30	1.250	3.550	1.050	0.602^a^	< 0.001^a^
12	I provide information about fertility preservation to patients before their cancer treatment.	2.65	1.300	3.950	1.350	0.848^a^	< 0.001^a^
13	The content of the information provided about fertility preservation is tailored to the patient's cancer condition and the status of their cancer treatment.	2.83	1.318	4.148	1.512	0.840^a^	< 0.001^a^
14	I provide information about fertility preservation, considering the patients' role in society.	3.06	1.179	4.239	1.881	0.656^a^	< 0.001^a^
15	I confirm whether the patient has anxiety and/or distrust regarding fertility preservation.	2.96	1.294	4.254	1.666	0.813^a^	< 0.001^a^
16	I provide information about fertility preservation after obtaining informed consent from the physician.	2.55	1.260	3.810	1.290	0.815^a^	< 0.001^a^
17	I choose the content of the information provided based on the presence of marriage.	2.56	1.287	3.847	1.273	0.792^a^	< 0.001^a^
18	I support the patient's emotional state, taking into account the background of their family.	3.54	1.012	4.552	2.528	0.606^a^	< 0.001^a^
19	I provide information about medical financial assistance to patients.	2.46	1.399	3.859	1.061	0.840^a^	< 0.001^a^
20	I am aware of the intentions of the patient's family and partner regarding fertility preservation.	2.69	1.283	3.973	1.407	0.783^a^	< 0.001^a^
21	I provide information about fertility preservation to the patient's family and partner.	2.33	1.185	3.515	1.145	0.852^a^	< 0.001^a^
22	I manage the wishes for fertility preservation when the patient's and their family/partner's wishes differ, ensuring that both the patient and family/partner agree.	2.93	1.162	4.092	1.768	0.581^a^	< 0.001^a^

SD, standard deviation; I-T relation, item-total correlations; GP analysis, good–poor analysis.

a *P* ​< ​0.001.

Based on the results of the scree plot and parallel analysis, a three-factor solution was adopted. The scree plot revealed a clear inflection point at the third factor, while the parallel analysis indicated that only the first three factors had eigenvalues exceeding those derived from randomly generated data. These findings support the statistical justification for retaining a three-factor model. Furthermore, the EFA revealed that the first three factors explained 36.0%, 19.2%, and 9.9% of the variance, respectively, yielding a cumulative explained variance of 65.1%. A three-factor structure accounting for 65.1% of the variance was confirmed (Table 3).

**Table 3:** Eigenvalues and percentage of variance explained in the exploratory factor analysis.

Factor	Eigenvalue	% of Variance explained	Cumulative %
1	7.449	35.985%	35.985%
2	3.970	19.174%	55.159%
3	2.049	9.894%	65.053%

**Fig. 2:**
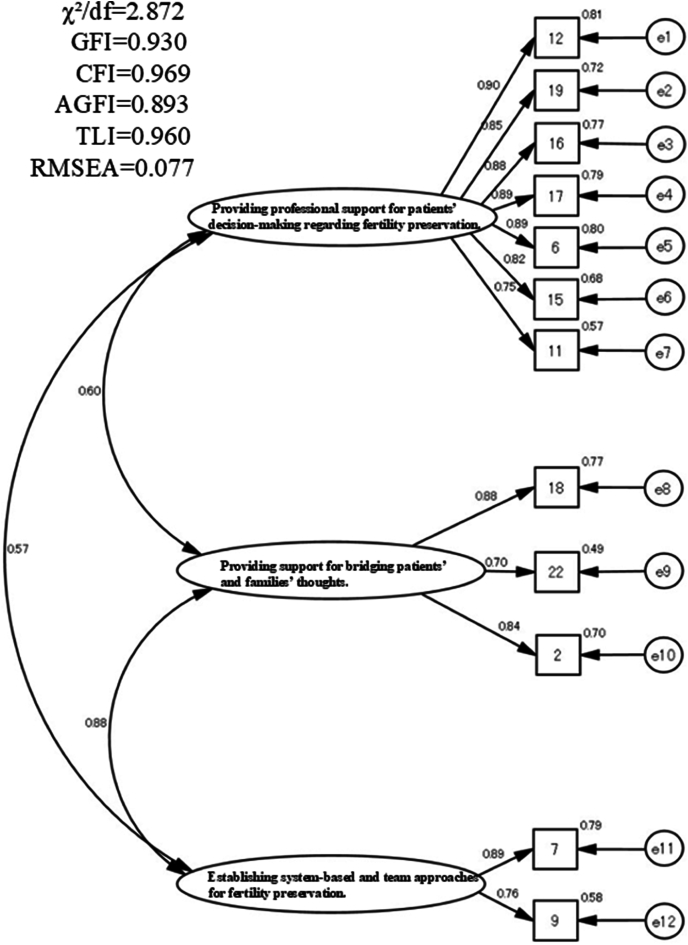
**Diagram of the confirmatory factor analysis model**. GFI, goodness of fit index; CFI, comparative fit index; AGFI, adjusted goodness of fit index; TLI, Tucker–Lewis index; RMSEA, root mean square error of approximation.

To examine the construct validity of the EFA, the value of the KMO measure of sampling adequacy was 0.914, which was significant (*P* ​< ​0.001). A three-factor model was selected based on the interpretability of the factors and the scree plot. Two items (#4 and #14) were excluded due to low value of factor loading (Supplementary Table S1) (0.374 and 0.279, respectively). Therefore, a three-factor scale comprising 12 items was created (Table 4). The first, second, and third factors identified were “providing professional support for the patient decision-making on FP” (seven items), “providing support for bridging patients' and families' thoughts” (three items), and “establishing a system and team approach for FP” (two items), respectively. For factors 1, 2, and 3, the Cronbach's α coefficient were 0.930, 0.846, and 0.772, respectively (overall: 0.921). Moreover, the Cronbach's α coefficients for all items were > 0.7. The correlations among the three factors ranged from *r* ​= ​0.669 to 0.424, and the cumulative contribution rate was 67.5%. For evaluating the model fit of the three-factor structure using CFA, the model for indices were χ^2^/df ​= ​2.872, CFI ​= ​0.969, TLI ​= ​0.960, and RMSEA ​= ​0.077 (*P* ​< ​0.001) (Fig. 2).

**Table 4:** Exploratory factor analysis findings.

No.	Items Total Cronbach's α coefficient ​= ​0.921	Factor 1	Factor 2	Factor 3
**Factor 1: Providing professional support for patients' decision-making regarding fertility preservation. (α** ​= ​**0.930, mean** ​= ​**18.08, SD** ​= ​**7.66)**				
12	I provide information about fertility preservation to patients before their cancer treatment.	0.907	−0.058	0.023
19	I provide information about medical financial assistance to patients.	0.906	−0.032	−0.016
16	I provide information about fertility preservation after obtaining informed consent from the physician.	0.900	−0.140	0.109
17	I choose the content of the information provided based on the presence of marriage.	0.851	−0.030	0.040
6	I confirm whether a difference exists between actual fertility preservation and the fertility preservation recognized by the patient.	0.774	0.206	−0.097
15	I confirm whether the patient has anxiety and/or distrust regarding fertility preservation.	0.677	0.314	−0.144
11	I share patient information regarding fertility preservation with the medical team involved in fertility preservation.	0.524	−0.069	0.128
**Factor 2: Providing support for bridging patients' and families' thoughts. (α** ​= ​**0.846, Mean** ​= ​**10.33, and SD** ​= ​**2.73)**				
18	I support the patient's emotional state, considering the background of their family.	−0.032	0.839	0.112
22	I manage the wishes for fertility preservation when the patient's and their family/partner's wishes differ, ensuring that both the patient and family/partner agree.	0.067	0.747	−0.035
2	I support the patient's emotional state, considering that the patient may be unstable and anxious.	−0.093	0.606	0.335
**Factor 3: Establishing system-based and team approaches for fertility preservation. (α** ​= ​**0.772, Mean** ​= ​**6.91, and SD** ​= ​**2.11)**				
7	I appropriately share patient information with the cancer specialist who is treating them.	0.145	0.074	0.714
9	I appropriately share patient information with the nurses who belong to the same department.	−0.015	0.080	0.706

**Factorial correlation matrix**				
Factor 1		–	0.558	0.424
Factor 2			–	0.669
Factor 3				–

SD, standard deviation; EFA, exploratory factor analysis.

The total scores on the scale for assessing nursing practice that support FP decision-making in women with cancer were significantly correlated with the Reflective Practice Scale for Nurses Regarding Patient Treatment Decision-Making (*r* ​= ​0.503, *P* ​< ​0.001) and its subscales (*r* ​= ​0.261–0.516, *P* ​< ​0.001) (Table 5). For convergent validity, the AVE values for factors 1, 2, and 3 were 0.732, 0.657, and 0.685, respectively. In contrast, the CR values for factors 1, 2, and 3 were 0.950, 0.850, and 0.812, respectively. Regarding discriminant validity, the AVE values for factors 1, 2, and 3 were 0.856, 0.810, and 0.828, respectively. Discriminant validity was further examined using the HTMT ratio of correlations. The HTMT values between factors 1 and 2 (0.673) and between factors 1 and 3 (0.634) were below the recommended threshold of 0.85, indicating adequate discriminant validity. However, the HTMT value between factors 2 and 3 was 0.917, suggesting potential conceptual overlap between them (Table 6).

**Table 5:** Criterion-related validity verification findings.

Scale for nursing to support patients' decision-making for treatments	Developed scale in this study			
	Entire Scale	Factor 1	Factor 2	Factor 3
Entire scale	0.503^a^	0.426^a^	0.527^a^	0.413^a^
Factor 1: Understanding the situation of the medical team	0.502^a^	0.432^a^	0.516^a^	0.397^a^
Factor 2: Recognition of nurses' role	0.389^a^	0.310^a^	0.444^a^	0.348^a^
Factor 3: Understanding the background of the patients and their family members	0.355^a^	0.307^a^	0.400^a^	0.261^a^
Factor 4: Understanding the situation of treatment	0.477^a^	0.407^a^	0.460^a^	0.406^a^
Factor 5: Deeply understanding the intentions of treatments in the patients and their family members	0.515^a^	0.431^a^	0.512^a^	0.415^a^

a *P* < 0.001.

**Table 6:** Convergent and discriminant validity indices, including HTMT ratios.

Measure	Factor 1	Factor 2	Factor 3
Inter-factor correlations	–	0.573^a^	0.442^a^
		–	0.667^a^
			–
AVE	0.732	0.657	0.685
Composite reliability	0.950	0.850	0.812
√AVE	0.856	0.810	0.828
HTMT rRatio (factor pairs)			
Factor 1	–	0.673	0.634
Factor 2		–	0.917
Factor 3			–

AVE, average variance extracted; √AVE, square root of the average variance extracted; HTMT, Heterotrait-Monotrait.

a *P* ​< ​0.001.

## Discussion

This study developed a scale to evaluate nursing practices that support FP decision-making in women with cancer. The scale comprised three factors and 12 items, and had good validity and reliability. Notably, the clinical implication of this scale lies in its ability to help healthcare providers recognize their strengths and gaps in support, which can improve the quality of decision-making. The scale may also contribute to the development of educational programs focused on oncofertility care.

### Scale characteristics

The items in the scale were developed based on a review of Japanese and English literature and discussions with nursing experts. According to the Consensus-Based Standards for the Selection of Health Measurement Instruments guideline framework,^41^ the scale included factors related to interventions designed to improve nursing practice that support FP decision-making in women with cancer.

Factor 1, “Providing professional support for patients' decision-making regarding fertility preservation,” reflected the importance of roles in nurses. Previous studies emphasize that decision-making regarding FP requires comprehensive, accurate, and timely information before initiating cancer treatment.^4^^,^^5^^,^^42^ The items under this factor include essential nursing practices, such as understanding patients' perspectives and providing fertility-related information, that consider patients' reproductive concerns and treatment context. This aligns with previous studies showing that professional support in shared decision-making enhances patients' autonomy and satisfaction,^43^, ^44^, ^45^ as well as with a qualitative research highlighting the importance of nurses' recognition of patients' reproductive values and decision preferences.^46^ Moreover, the scale developed in this study may also include the essential content related to nurses' recognition of patients’ decision-making.

Factor 2, “Providing support for bridging patients' and families' thoughts,” encompasses the relational and emotional dimensions of decision-making. A previous study reported that decision-making regarding FP were affected by psychological responses and understanding from the family and/or partner.^16^ FP decision is also significantly affected by personal values related to family planning and reproduction^17^ in the future, which supports the findings of this study. Previous studies have shown that psychological responses in patients, along with the understanding and cooperation of their families and/or partners, significantly influenced decision-making.^47^^,^^48^ The findings of these previous studies align with ours. FP decisions usually depend on the patient's values, as well as the agreement and understanding of their families. Nurses play an important role in mediating between the patient and their family when decisions for FP are conflicting.^49^ By facilitating mutual understanding and providing psychosocial support, nurses help patients navigate value-laden decisions in a limited timeframe, typically under stress.^50^^,^^51^ This support can help patients feel that “the nurse truly understands my thoughts,” which might correlate with satisfaction with the decisions and positive motivation toward treatment. The present scale uniquely highlights nurses' role in mediating between the patient and family, an aspect that is usually underrepresented in patient-centered care approaches. This relational focus is particularly relevant in cultures such as Japan, where family members are usually involved in healthcare decision-making.

Factor 3, “Establishing system-based and team approaches for fertility preservation,” highlights the importance of team-based healthcare in supporting FP decision-making for women with cancer. Interdisciplinary collaboration and appropriate information-sharing are critical to ensure timely and consistent support. Additionally, individualized support is enabled when nurses accurately communicate patient preferences and clinical context to specialists, which is consistent with the findings of the present study.^52^ Information sharing among nurses within departments is essential to maintain continuous and consistent nursing practices. Decision-making support for FP should not be the responsibility of a single medical profession.^53^ Inadequate information sharing among the medical team members during the process can lead to difficulty in providing timely support to patients’ needs, an observation that agrees with the findings of the present study.^42^^,^^54^ These findings indicated that the structure of “information sharing” and “team-based approaches” form the foundation for informed and satisfactory patient decision-making. The three-factor structure in this study might include a minimum essential set of nursing practices supporting FP decision-making. Previous studies have focused on aspects such as information provision and emotional support.^16^^,^^17^^,^^50^^,^^51^ However, the current scale integrates these components within a comprehensive model that includes bridging patients/family and their collaboration. This suggests that effective nursing support requires a holistic and coordinated approach, extending beyond individual competencies to systemic and team-based functions.

### Theoretical and practical implications

This scale encompasses key domains of nursing practice and the Minimum Data Set, which is defined as the core elements necessary to evaluate and improve support for FP decision-making. It can serve as a benchmark for assessing the implementation of SDM-based care and a foundation for designing educational interventions for nurses. Therefore, the scale has strong potential for practical application in clinical settings, as well as for research and educational purposes.

### Examination of the Scale's reliability and validity

Based on the EFA results, the items in the newly developed scale to evaluate nursing practice supporting FP decision-making in women with cancer were refined from 22 to 12 across the three factors. The Cronbach's α coefficient was above 0.7 for all factors in this study, indicating that they had reliability.^36^ CFI, RMSEA, and TLI were > 0.90, < 0.08, and > 0.90, respectively, indicating that all met the criteria for an acceptable model fit. This study also showed an AVE of ≥ 0.66 and a CR of ≥ 0.81, both of which exceeded the threshold (AVE > 0.50 and CR ​> ​0.70). For each factor, √AVE was greater than its correlations with other factors, indicating that discriminant validity was also established according to Fornell and Larcker's criterion.^40^ However, the correlation between factors 2 and 3 was relatively high (*r* ​= ​0.667), and √AVE for these two factors were higher than the inter-factor correlation (√AVE_2_ ​= ​0.810 and √AVE_3_ ​= ​0.828), suggesting that potential conceptual overlap exists. To further address this, we conducted HTMT ratio analysis. The HTMT value between factors 2 and 3 was 0.917, exceeding the generally recommended threshold of 0.90. This finding indicates that the distinction between factors 2 and 3 is insufficiently strong, while discriminant validity was mostly supported. Furthermore, this may reflect the conceptual closeness of these domains in real-world nursing practice, where nurses' recognition of their professional roles (factor 2) is usually closely related to understanding the background of patients and their families (factor 3). Despite this overlap, the overall construct validity of the scale is considered acceptable.

Moreover, these results suggest that the scale has good construct validity. For criterion-related validity, we hypothesized that nursing practices supporting FP decision-making might be correlated with general nursing practices (i.e., the Reflective Practice Scale for Nurses Regarding Patient Treatment Decision-Making). These results indicate that FP decision-making in women with cancer is more proactively supported by nurses with high nursing skills in treatment decision-making. However, in our study, the scale was developed based on the SDM model, where the components of patient engagement, information sharing, and collaborative decision-making are conceptually interrelated. The moderate to high correlations observed were considered theoretically justifiable because of the differences in the underlying model. Overall, the findings from the EFA, CFA, construct validity, and criterion-related validity indicated that the developed scale is suitable for assessing nursing practices supporting FP decision-making in women with cancer.

### Limitations and future directions

This study has some limitations. First, participants were categorized into two groups for EFA and CFA, and data were obtained during different periods. Although the recruitment methods and eligibility criteria were similar, significant differences were found between the groups. Specifically, the EFA group included a higher proportion of participants with experience in oncofertility counseling and relevant qualifications than the CFA group. These differences may have acted as confounding factors, potentially affecting the results’ generalizability. To reduce such biases, future research should consider matching participants based on background characteristics or adopting stratified sampling techniques.

Second, the study employed convenience sampling due to the limited availability of eligible participants during the study period. While this approach enabled efficient data collection, it may have limited the sample's representativeness. Furthermore, this study was conducted in Japan; therefore, the findings may not be generalizable to nurses in other countries, where differences in nurses' roles in fertility care, the availability of FP services, and societal attitudes toward cancer and fertility could influence the developed scale's applicability. Future studies should adopt more rigorous sampling strategies and include larger, more diverse, and internationally representative samples to examine the scale's cross-cultural validity and relevance.

Third, the possibility of selection bias should be considered. Nurses with a greater interest in FP support may have been more likely to participate, potentially leading to an overestimation of the extent of such practices. Although the response rates for the EFA and CFA were 34.5% and 41.1%, respectively—comparable to those reported in other studies targeting healthcare professionals—they were relatively low, raising the possibility of non-response bias. Since the survey was conducted anonymously, we could not compare the characteristics of respondents and non-respondents, which may further limit the findings' generalizability. Finally, this study did not include women with cancer who needed support regarding FP, which may limit the reflection of patients’ concerns about FP. Despite this limitation, no scale for evaluating nursing practices that support FP decision-making in women with cancer exists to date. We believe further studies should include perspectives from both nurses and patients. Furthermore, this study did not include the time course factor, which can affect changes in concerns and needs among women with cancer.

## Conclusions

This study showed that the developed scale, comprising three factors and 12 items, is both reliable and valid for assessing nursing practices that support FP decision-making in women with cancer. The scale has potential utility in facilitating FP decision-making in women with cancer within clinical nursing practice.

## CRediT authorship contribution statement

Mikako Yoshihara: Conceptualization, Methodology, Formal analysis and investigation, Writing - Original Draft Preparation, Funding Acquisition, Resources. Kazuaki Tanabe: Conceptualization, Methodology, Writing - Review and Editing, Supervision. Hisae Nakatani: Conceptualization, Methodology, Formal analysis and investigation, Writing - Review and Editing, Supervision. Ruixin Lei: Conceptualization, Formal analysis and investigation, Writing - Review and Editing. Hiroyuki Sawatari: Methodology, Formal analysis and investigation, Writing - Review and Editing. Chie Teramoto: Methodology, Writing -Review and Editing. All authors have read and approved the final manuscript.

## Ethics statement

This study was approved by the Epidemiological Research Ethics Committee of Hiroshima University (Approval No. E2022-0146) and was conducted in accordance with the 1964 Helsinki Declaration and its later amendments or comparable ethical standards. All participants provided written informed consent.

## Data availability statement

The data supporting the findings of this study are available from the corresponding author, KT, upon request.

## Declaration of generative AI and AI-assisted technologies in the writing process

No AI tools were used in the writing process of this study.

## Funding

This study was supported by The Yasuda Medical Foundation (Grant No. 2022Y-22). The funders had no role in considering the study design or in the collection, analysis, interpretation of data, writing of the report, or decision to submit the article for publication.

## Declaration of competing interest

The authors declare that they have no competing interests.
