# Supplementary material for: Development and validation of a nursing practice scale for supporting fertility preservation decision-making in women with cancer
Source: Asia Pac J Oncol Nurs. 2025 Oct 24;12:100808. doi: 10.1016/j.apjon.2025.100808 (PMC12720104; doi:10.1016/j.apjon.2025.100808)
Supplement: Multimedia component 1 [file mmc1.docx]

**Supplementary Table S1. Factor loadings of all scale items (including dropped items #4 and #14)**

| No. | **Items** | Factor 1 | Factor 2 | Factor 3 | Status |
| --- | --- | --- | --- | --- | --- |
| 12 | I provide information about fertility preservation to patients before their cancer treatment. | 0.907 | -0.058 | 0.023 | Retained |
| 19 | I provide information about medical financial assistance to patients. | 0.906 | -0.032 | -0.016 | Retained |
| 16 | I provide information about fertility preservation after obtaining informed consent from the physician. | 0.900 | -0.140 | 0.109 | Retained |
| 17 | I choose the content of the information provided based on the presence of marriage. | 0.851 | -0.030 | 0.040 | Retained |
| 6 | I confirm whether a difference exists between actual fertility preservation and the fertility preservation recognized by the patient. | 0.774 | 0.206 | -0.097 | Retained |
| 15 | I confirm whether the patient has anxiety and/or distrust regarding fertility preservation. | 0.677 | 0.314 | -0.144 | Retained |
| 11 | I share patient information regarding fertility preservation with the medical team involved in fertility preservation. | 0.524 | -0.069 | 0.128 | Retained |
| 4 | I gather information about the presence of the patient’s partner and their relationship early after the cancer diagnosis. | 0.374 | 0.110 | 0.262 | Dropped |
| 14 | I provide information about fertility preservation, considering the patients’ role in society. | 0.279 | 0.001 | 0.128 | Dropped |
| 18 | I support the patient’s emotional state, considering the background of their family. | -0.032 | 0.839 | 0.112 | Retained |
| 22 | I manage the wishes for fertility preservation when the patient’s and their family/partner’s wishes differ, ensuring that both the patient and family/partner agree. | 0.067 | 0.747 | -0.035 | Retained |
| 2 | I support the patient’s emotional state, considering that the patient may be unstable and anxious. | -0.093 | 0.606 | 0.335 | Retained |
| 7 | I appropriately share patient information with the cancer specialist who is treating them. | 0.145 | 0.074 | 0.714 | Retained |
| 9 | I appropriately share patient information with the nurses who belong to the same department. | -0.015 | 0.080 | 0.706 | Retained |

*Note: Loadings below 0.40 were considered insufficient, and items #4 and #14 were excluded from the final scale.*
